# *N*-(Biphenyl-4-ylcarbon­yl)-*N*′-(2-pyridylmeth­yl)thio­urea

**DOI:** 10.1107/S1600536807067499

**Published:** 2008-01-04

**Authors:** Bohari M. Yamin, Hidayah Deris, Zaw Myint Malik, Sammer Yousuf

**Affiliations:** a School of Chemical Sciences and Food Technology, Universiti Kebangsaan Malaysia, UKM 43600 Bangi Selangor, Malaysia; b HEJ Research Institute of Chemistry, University of Karachi, Karachi 75270, Pakistan

## Abstract

In the title compound, C_20_H_17_N_3_OS, the dihedral angle between the benzene rings of the biphenyl fragment is 36.84 (9)°. The *trans*–*cis* geometry of the thio­urea unit is stabilized by intra­molecular N—H⋯O and N—H⋯N hydrogen bonds between the H atom of the *cis* thio­amide and the carbonyl O and pyridine N atoms, respectively. In the crystal structure, inter­molecular N—H⋯S hydrogen bonds form centrosymmetric dimers extending along the *b* axis.

## Related literature

For the crystal structure of the biphenyl-4-carbonyl­thio­urea analogue, see: Arif & Yamin (2007 ▶). For bond-length data, see: Allen *et al.* (1987 ▶).
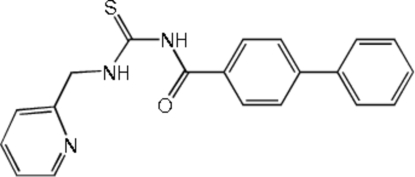


## Experimental

### 

#### Crystal data

C_20_H_17_N_3_OS
*M* *_r_* = 347.43
Triclinic, 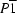

*a* = 7.467 (2) Å
*b* = 9.364 (2) Å
*c* = 13.184 (3) Å
α = 101.529 (5)°
β = 99.113 (4)°
γ = 101.543 (5)°
*V* = 865.9 (4) Å^3^
*Z* = 2
Mo *K*α radiation
μ = 0.20 mm^−1^
*T* = 273 (2) K
0.45 × 0.37 × 0.18 mm

#### Data collection

Bruker SMART APEX CCD area-detector diffractometer
Absorption correction: multi-scan (*SADABS*; Bruker, 2000 ▶) *T*_min_ = 0.915, *T*_max_ = 0.965
8243 measured reflections
3036 independent reflections
2561 reflections with *I* > 2σ(*I*)
*R*_int_ = 0.018

#### Refinement

*R*[*F* ^2^ > 2σ(*F* ^2^)] = 0.037
*wR*(*F* ^2^) = 0.101
*S* = 1.05
3036 reflections
226 parameters
H-atom parameters constrained
Δρ_max_ = 0.20 e Å^−3^
Δρ_min_ = −0.19 e Å^−3^

### 

Data collection: *SMART* (Bruker, 2000 ▶); cell refinement: *SAINT* (Bruker, 2000 ▶); data reduction: *SAINT*; program(s) used to solve structure: *SHELXS97* (Sheldrick, 1997*a* ▶); program(s) used to refine structure: *SHELXL97* (Sheldrick, 1997*a* ▶); molecular graphics: *SHELXTL* (Sheldrick, 1997*b* ▶); software used to prepare material for publication: *SHELXTL*, *PARST* (Nardelli, 1995 ▶) and *PLATON* (Spek, 2003 ▶).

## Supplementary Material

Crystal structure: contains datablocks global, I. DOI: 10.1107/S1600536807067499/at2519sup1.cif

Structure factors: contains datablocks I. DOI: 10.1107/S1600536807067499/at2519Isup2.hkl

Additional supplementary materials:  crystallographic information; 3D view; checkCIF report

## Figures and Tables

**Table 1:** Hydrogen-bond geometry (Å, °)

*D*—H⋯*A*	*D*—H	H⋯*A*	*D*⋯*A*	*D*—H⋯*A*
N2—H2⋯O1	0.86	1.99	2.6681 (19)	135
N2—H2⋯N3	0.86	2.24	2.6488 (19)	109
N1—H1⋯S1^i^	0.86	2.79	3.4759 (17)	138

Symmetry code: (i) 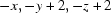.

**Fig. 1.**
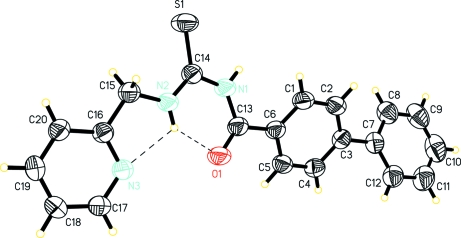
The molecular structure of (I), with displacement ellipsods are drawn at the 50% probability level.

**Fig. 2.**
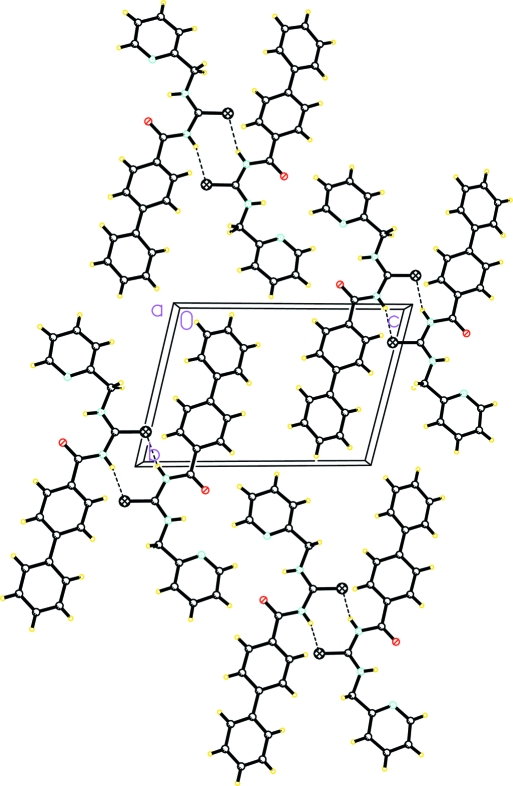
A packing diagram of (I) viewed down the a axis. Hydrogen bonds are shown by dashed lines.

## supplementary crystallographic information

### Comment

The title compound, (I), analogous to *N*-(biphenyl-4-carbonyl)-N'- (2-chlorophenyl)thiourea (II) (Arif & Yamin, 2007) except the 2-chlorobenzene group is replaced by the 2-methyl-pyridine group (Fig.1). The molecule maintains its *trans*-*cis* configuration with respect to the position of the biphenyl-4-carbonyl and 2-methyl-pyridin groups relative the thiono sulfur atom across the C14—N1 and C14—N2 bonds, respectively. Other bond lengths and angles are in normal ranges (Allen *et al.*, 1987) and comparable to those in (II). However, the dihedral angle between the benzene rings of the biphenyl fragment, (C1—C6) and (C7—C12) of 36,84 (9)° is larger than that (20.71 (17)°) in (II). Both the central C13/O1/N1/C14/S1/N2/C15 fragment and pyridine ring (N3/C16—C20), are planar with a maximum deviation of 0.032 (2)Å for atom N1 atom from the least square plane of the central fragment. The central fragment makes dihedral angles with the (C7—C12) benzene and (N3/C16—C20) pyridine rings of 16.39 (8) and 13.21 (6)°, respectively. The *trans*-*cis* geometry of the thiourea moiety is stabilized by the relatively strong N2—H2···O1 and a weak N2—H2···N3 intramolecular hydrogen bonds (Table 2). In the crystal structure, the molecules are linked by N1—H1···S1 intermolecular hydrogen bonds to form centrosymmetric dimers and are arranged parallel to *b* axis (Fig.2). In (II), the molecule is stabilized by van der Waal and π-π interactions.

### Experimental

The mixrure of biphenyl 1–4 carbonyl chloride (5.417 g, 0.025 mol), with the equimolar amount of ammonium thiocyanate (1.903 g, 0.025 mol) and 2-picolylamine (2.704 g, 0.025 mol) in 30 ml dry acetone was refluxed with stirring for 4 h. The solution was filtered and left to evaporate at room temperature. The black precipitate obtained after a few days, was washed with water and cold ethanol (80%; m.p 416.4–419.2 K). Suitable crystals for X-ray investigation were obtained byrecrystallization from mixture of dichloromethane and n-Hexane (1:3 *v*/*v*).

### Refinement

H atoms on both the C and N atoms were positioned geometrically with C—H = 0.93 - 0.97Å and N—H = 0.86Å and constrained to ride on their parent atoms with *U*_iso_(H)= 1.2*U*_eq_(parent atom).

### Crystal data

**Table:**

C_20_H_17_N_3_OS	*Z* = 2
*M**_r_* = 347.43	*F*_000_ = 364
Triclinic, *P*1	*D*_x_ = 1.333 Mg m^−^^3^
Hall symbol: -P 1	Mo *K*α radiation λ = 0.71073 Å
*a* = 7.467 (2) Å	Cell parameters from 3740 reflections
*b* = 9.364 (2) Å	θ = 1.6–25.0º
*c* = 13.184 (3) Å	µ = 0.20 mm^−^^1^
α = 101.529 (5)º	*T* = 273 (2) K
β = 99.113 (4)º	Block, colourless
γ = 101.543 (5)º	0.45 × 0.37 × 0.18 mm
*V* = 865.9 (4) Å^3^	

### Data collection

**Table:**

Bruker SMART APEX CCD area-detector diffractometer	3036 independent reflections
Radiation source: fine-focus sealed tube	2561 reflections with *I* > 2σ(*I*)
Monochromator: graphite	*R*_int_ = 0.019
Detector resolution: 83.66 pixels mm^-1^	θ_max_ = 25.0º
*T* = 273(2) K	θ_min_ = 1.6º
ω scans	*h* = −8→8
Absorption correction: multi-scan(SADABS; Bruker, 2000)	*k* = −11→11
*T*_min_ = 0.915, *T*_max_ = 0.965	*l* = −15→15
8243 measured reflections	

### Refinement

**Table:**

Refinement on *F*^2^	Secondary atom site location: difference Fourier map
Least-squares matrix: full	Hydrogen site location: inferred from neighbouring sites
*R*[*F*^2^ > 2σ(*F*^2^)] = 0.037	H-atom parameters constrained
*wR*(*F*^2^) = 0.101	*w* = 1/[σ^2^(*F*_o_^2^) + (0.0535*P*)^2^ + 0.1413*P*] where *P* = (*F*_o_^2^ + 2*F*_c_^2^)/3
*S* = 1.05	(Δ/σ)_max_ < 0.001
3036 reflections	Δρ_max_ = 0.20 e Å^−^^3^
226 parameters	Δρ_min_ = −0.19 e Å^−^^3^
Primary atom site location: structure-invariant direct methods	Extinction correction: none

### Special details

**Table:**

Geometry. All e.s.d.'s (except the e.s.d. in the dihedral angle between two l.s. planes) are estimated using the full covariance matrix. The cell e.s.d.'s are taken into account individually in the estimation of e.s.d.'s in distances, angles and torsion angles; correlations between e.s.d.'s in cell parameters are only used when they are defined by crystal symmetry. An approximate (isotropic) treatment of cell e.s.d.'s is used for estimating e.s.d.'s involving l.s. planes.
Refinement. Refinement of *F*^2^ against ALL reflections. The weighted *R*-factor *wR* and goodness of fit *S* are based on *F*^2^, conventional *R*-factors *R* are based on *F*, with *F* set to zero for negative *F*^2^. The threshold expression of *F*^2^ > σ(*F*^2^) is used only for calculating *R*-factors(gt) *etc*. and is not relevant to the choice of reflections for refinement. *R*-factors based on *F*^2^ are statistically about twice as large as those based on *F*, and *R*- factors based on ALL data will be even larger.

### Fractional atomic coordinates and isotropic or equivalent isotropic displacement parameters (Å^2^)

**Table:**

	*x*	*y*	*z*	*U*_iso_*/*U*_eq_	
S1	0.04886 (8)	0.78345 (5)	1.01142 (3)	0.06855 (19)	
O1	0.0915 (2)	0.84641 (13)	0.68251 (9)	0.0672 (4)	
N1	0.1098 (2)	0.91996 (14)	0.85943 (10)	0.0523 (4)	
H1	0.1330	0.9985	0.9106	0.063*	
N2	0.00553 (19)	0.66245 (13)	0.80744 (10)	0.0481 (3)	
H2	0.0145	0.6723	0.7448	0.058*	
N3	−0.09400 (19)	0.44728 (14)	0.63072 (10)	0.0488 (3)	
C1	0.3283 (3)	1.20743 (18)	0.85384 (13)	0.0578 (4)	
H1A	0.3503	1.1786	0.9173	0.069*	
C2	0.4042 (3)	1.35237 (19)	0.84981 (13)	0.0570 (4)	
H2A	0.4774	1.4198	0.9109	0.068*	
C3	0.3741 (2)	1.40021 (17)	0.75677 (12)	0.0477 (4)	
C4	0.2679 (2)	1.29480 (18)	0.66663 (13)	0.0500 (4)	
H4	0.2473	1.3231	0.6029	0.060*	
C5	0.1927 (2)	1.14970 (18)	0.66996 (12)	0.0497 (4)	
H5	0.1232	1.0811	0.6084	0.060*	
C6	0.2193 (2)	1.10431 (17)	0.76401 (12)	0.0476 (4)	
C7	0.4562 (2)	1.55747 (18)	0.75548 (13)	0.0515 (4)	
C8	0.4688 (3)	1.6749 (2)	0.84207 (15)	0.0643 (5)	
H8	0.4199	1.6550	0.8998	0.077*	
C9	0.5536 (3)	1.8209 (2)	0.84273 (18)	0.0762 (6)	
H9	0.5616	1.8986	0.9009	0.091*	
C10	0.6255 (3)	1.8512 (2)	0.7583 (2)	0.0830 (7)	
H10	0.6842	1.9491	0.7595	0.100*	
C11	0.6113 (3)	1.7377 (2)	0.67184 (19)	0.0781 (6)	
H11	0.6591	1.7591	0.6141	0.094*	
C12	0.5262 (2)	1.5910 (2)	0.66975 (15)	0.0607 (5)	
H12	0.5162	1.5147	0.6104	0.073*	
C13	0.1346 (2)	0.94586 (17)	0.76308 (12)	0.0500 (4)	
C14	0.0520 (2)	0.78342 (16)	0.88501 (12)	0.0471 (4)	
C15	−0.0600 (3)	0.51329 (16)	0.82179 (12)	0.0506 (4)	
H15A	−0.1582	0.5129	0.8619	0.061*	
H15B	0.0418	0.4841	0.8615	0.061*	
C16	−0.1336 (2)	0.40249 (16)	0.71635 (12)	0.0433 (3)	
C17	−0.1583 (2)	0.34846 (18)	0.53719 (13)	0.0531 (4)	
H17	−0.1312	0.3784	0.4771	0.064*	
C18	−0.2621 (2)	0.20551 (18)	0.52501 (13)	0.0556 (4)	
H18	−0.3042	0.1403	0.4584	0.067*	
C19	−0.3025 (2)	0.16096 (18)	0.61372 (14)	0.0571 (4)	
H19	−0.3731	0.0648	0.6080	0.069*	
C20	−0.2370 (2)	0.26031 (17)	0.71097 (13)	0.0504 (4)	
H20	−0.2619	0.2322	0.7721	0.060*	

### Atomic displacement parameters (Å^2^)

**Table:**

	*U*^11^	*U*^22^	*U*^33^	*U*^12^	*U*^13^	*U*^23^
S1	0.1197 (5)	0.0465 (3)	0.0393 (3)	0.0166 (3)	0.0220 (2)	0.00870 (19)
O1	0.1052 (10)	0.0460 (7)	0.0428 (6)	0.0006 (6)	0.0275 (6)	0.0016 (5)
N1	0.0788 (9)	0.0363 (7)	0.0391 (7)	0.0075 (6)	0.0201 (6)	0.0027 (5)
N2	0.0685 (9)	0.0363 (7)	0.0378 (7)	0.0080 (6)	0.0155 (6)	0.0060 (5)
N3	0.0621 (8)	0.0403 (7)	0.0430 (7)	0.0081 (6)	0.0142 (6)	0.0092 (6)
C1	0.0791 (12)	0.0500 (9)	0.0405 (9)	0.0038 (8)	0.0147 (8)	0.0118 (7)
C2	0.0716 (11)	0.0478 (9)	0.0434 (9)	0.0012 (8)	0.0108 (8)	0.0064 (7)
C3	0.0480 (9)	0.0461 (8)	0.0515 (9)	0.0119 (7)	0.0155 (7)	0.0127 (7)
C4	0.0560 (9)	0.0520 (9)	0.0452 (9)	0.0150 (8)	0.0118 (7)	0.0157 (7)
C5	0.0560 (9)	0.0473 (9)	0.0431 (9)	0.0114 (7)	0.0100 (7)	0.0055 (7)
C6	0.0575 (9)	0.0431 (8)	0.0427 (8)	0.0099 (7)	0.0183 (7)	0.0071 (7)
C7	0.0473 (9)	0.0477 (9)	0.0595 (10)	0.0104 (7)	0.0059 (7)	0.0178 (8)
C8	0.0716 (12)	0.0499 (10)	0.0671 (12)	0.0104 (9)	0.0075 (9)	0.0141 (9)
C9	0.0860 (14)	0.0482 (10)	0.0823 (15)	0.0106 (10)	−0.0078 (11)	0.0131 (10)
C10	0.0821 (14)	0.0581 (12)	0.0998 (17)	−0.0004 (10)	−0.0113 (12)	0.0394 (13)
C11	0.0773 (14)	0.0774 (14)	0.0834 (15)	0.0063 (11)	0.0097 (11)	0.0457 (13)
C12	0.0610 (10)	0.0598 (11)	0.0637 (11)	0.0116 (8)	0.0097 (9)	0.0257 (9)
C13	0.0611 (10)	0.0440 (9)	0.0441 (9)	0.0086 (7)	0.0191 (7)	0.0065 (7)
C14	0.0577 (9)	0.0391 (8)	0.0437 (9)	0.0104 (7)	0.0149 (7)	0.0063 (7)
C15	0.0687 (10)	0.0393 (8)	0.0433 (9)	0.0096 (7)	0.0140 (8)	0.0102 (7)
C16	0.0508 (9)	0.0384 (8)	0.0426 (8)	0.0134 (7)	0.0130 (7)	0.0088 (6)
C17	0.0648 (10)	0.0510 (9)	0.0412 (9)	0.0090 (8)	0.0139 (7)	0.0084 (7)
C18	0.0629 (10)	0.0487 (9)	0.0456 (9)	0.0049 (8)	0.0073 (8)	0.0008 (7)
C19	0.0626 (10)	0.0407 (8)	0.0597 (10)	−0.0011 (8)	0.0114 (8)	0.0078 (8)
C20	0.0604 (10)	0.0442 (9)	0.0476 (9)	0.0089 (7)	0.0161 (7)	0.0133 (7)

### Geometric parameters (Å, °)

**Table:**

S1—C14	1.6703 (16)	C7—C12	1.383 (2)
O1—C13	1.2147 (18)	C7—C8	1.394 (2)
N1—C13	1.3735 (19)	C8—C9	1.385 (3)
N1—C14	1.390 (2)	C8—H8	0.9300
N1—H1	0.8600	C9—C10	1.365 (3)
N2—C14	1.3102 (19)	C9—H9	0.9300
N2—C15	1.4449 (19)	C10—C11	1.368 (3)
N2—H2	0.8600	C10—H10	0.9300
N3—C16	1.3351 (19)	C11—C12	1.386 (3)
N3—C17	1.336 (2)	C11—H11	0.9300
C1—C2	1.377 (2)	C12—H12	0.9300
C1—C6	1.386 (2)	C15—C16	1.505 (2)
C1—H1A	0.9300	C15—H15A	0.9700
C2—C3	1.389 (2)	C15—H15B	0.9700
C2—H2A	0.9300	C16—C20	1.381 (2)
C3—C4	1.391 (2)	C17—C18	1.370 (2)
C3—C7	1.482 (2)	C17—H17	0.9300
C4—C5	1.375 (2)	C18—C19	1.374 (2)
C4—H4	0.9300	C18—H18	0.9300
C5—C6	1.387 (2)	C19—C20	1.375 (2)
C5—H5	0.9300	C19—H19	0.9300
C6—C13	1.489 (2)	C20—H20	0.9300
			
C13—N1—C14	128.47 (13)	C9—C10—H10	119.9
C13—N1—H1	115.8	C11—C10—H10	119.9
C14—N1—H1	115.8	C10—C11—C12	120.4 (2)
C14—N2—C15	123.28 (13)	C10—C11—H11	119.8
C14—N2—H2	118.4	C12—C11—H11	119.8
C15—N2—H2	118.4	C7—C12—C11	120.35 (19)
C16—N3—C17	117.48 (13)	C7—C12—H12	119.8
C2—C1—C6	120.47 (15)	C11—C12—H12	119.8
C2—C1—H1A	119.8	O1—C13—N1	122.47 (14)
C6—C1—H1A	119.8	O1—C13—C6	122.08 (14)
C1—C2—C3	121.59 (15)	N1—C13—C6	115.45 (13)
C1—C2—H2A	119.2	N2—C14—N1	117.11 (13)
C3—C2—H2A	119.2	N2—C14—S1	124.42 (12)
C2—C3—C4	117.39 (14)	N1—C14—S1	118.47 (11)
C2—C3—C7	120.20 (14)	N2—C15—C16	110.45 (12)
C4—C3—C7	122.41 (14)	N2—C15—H15A	109.6
C5—C4—C3	121.30 (14)	C16—C15—H15A	109.6
C5—C4—H4	119.4	N2—C15—H15B	109.6
C3—C4—H4	119.4	C16—C15—H15B	109.6
C4—C5—C6	120.79 (15)	H15A—C15—H15B	108.1
C4—C5—H5	119.6	N3—C16—C20	122.55 (14)
C6—C5—H5	119.6	N3—C16—C15	117.53 (13)
C1—C6—C5	118.42 (14)	C20—C16—C15	119.92 (13)
C1—C6—C13	123.02 (14)	N3—C17—C18	123.61 (15)
C5—C6—C13	118.53 (14)	N3—C17—H17	118.2
C12—C7—C8	118.43 (16)	C18—C17—H17	118.2
C12—C7—C3	120.95 (16)	C17—C18—C19	118.35 (15)
C8—C7—C3	120.60 (15)	C17—C18—H18	120.8
C9—C8—C7	120.47 (19)	C19—C18—H18	120.8
C9—C8—H8	119.8	C18—C19—C20	119.16 (15)
C7—C8—H8	119.8	C18—C19—H19	120.4
C10—C9—C8	120.2 (2)	C20—C19—H19	120.4
C10—C9—H9	119.9	C19—C20—C16	118.86 (15)
C8—C9—H9	119.9	C19—C20—H20	120.6
C9—C10—C11	120.11 (19)	C16—C20—H20	120.6
			
C6—C1—C2—C3	0.3 (3)	C14—N1—C13—O1	6.5 (3)
C1—C2—C3—C4	−1.6 (3)	C14—N1—C13—C6	−173.11 (16)
C1—C2—C3—C7	179.29 (16)	C1—C6—C13—O1	−156.33 (17)
C2—C3—C4—C5	1.2 (2)	C5—C6—C13—O1	21.6 (2)
C7—C3—C4—C5	−179.76 (15)	C1—C6—C13—N1	23.3 (2)
C3—C4—C5—C6	0.6 (2)	C5—C6—C13—N1	−158.76 (15)
C2—C1—C6—C5	1.5 (3)	C15—N2—C14—N1	−178.94 (15)
C2—C1—C6—C13	179.51 (16)	C15—N2—C14—S1	1.9 (2)
C4—C5—C6—C1	−2.0 (2)	C13—N1—C14—N2	−3.7 (3)
C4—C5—C6—C13	179.95 (15)	C13—N1—C14—S1	175.48 (14)
C2—C3—C7—C12	141.85 (17)	C14—N2—C15—C16	170.01 (14)
C4—C3—C7—C12	−37.2 (2)	C17—N3—C16—C20	−0.1 (2)
C2—C3—C7—C8	−36.5 (2)	C17—N3—C16—C15	179.56 (14)
C4—C3—C7—C8	144.45 (17)	N2—C15—C16—N3	13.4 (2)
C12—C7—C8—C9	−1.5 (3)	N2—C15—C16—C20	−166.90 (14)
C3—C7—C8—C9	176.90 (16)	C16—N3—C17—C18	0.2 (2)
C7—C8—C9—C10	0.1 (3)	N3—C17—C18—C19	0.0 (3)
C8—C9—C10—C11	1.1 (3)	C17—C18—C19—C20	−0.3 (3)
C9—C10—C11—C12	−0.8 (3)	C18—C19—C20—C16	0.5 (3)
C8—C7—C12—C11	1.8 (3)	N3—C16—C20—C19	−0.2 (2)
C3—C7—C12—C11	−176.64 (16)	C15—C16—C20—C19	−179.91 (15)
C10—C11—C12—C7	−0.6 (3)		

### Hydrogen-bond geometry (Å, °)

**Table:**

*D*—H···*A*	*D*—H	H···*A*	*D*···*A*	*D*—H···*A*
N2—H2···O1	0.86	1.99	2.6681 (19)	135
N2—H2···N3	0.86	2.24	2.6488 (19)	109
N1—H1···S1^i^	0.86	2.79	3.4759 (17)	138

Symmetry codes:  (i) −*x*, −*y*+2, −*z*+2.

## References

[bb1] Allen, F. H., Kennard, O., Watson, D. G., Brammer, L., Orpen, A. G. & Taylor, R. (1987). *J. Chem. Soc. Perkin Trans. 2*, pp. S1–19.

[bb2] Arif, M. A. M. & Yamin, B. M. (2007). *Acta Cryst.* E**63**, o3594. 10.1107/S1600536807062265 PMC2915178 21200669

[bb3] Bruker (2000). *SADABS* (Version 2.01), *SMART* (Version 5.630) and *SAINT* (Version 6.36a). Bruker AXS Inc., Madison, Wisconsin, USA.

[bb4] Nardelli, M. (1995). *J. Appl. Cryst.* **28**, 659.

[bb5] Sheldrick, G. M. (1997*a*). *SHELXS97* and *SHELXL97*, University of Göttingen, Germany.

[bb6] Sheldrick, G. M. (1997*b*). *SHELXTL* Version 5.1. Bruker AXS, Inc., Madison, Wisconsin, USA.

[bb7] Spek, A. L. (2003). *J. Appl. Cryst.* **36**, 7–13.

